# An avian influenza virus A(H7N9) reassortant that recently emerged in the United States with low pathogenic phenotype does not efficiently infect swine

**DOI:** 10.1111/irv.12631

**Published:** 2019-02-13

**Authors:** Joshua D. Powell, Eugenio J. Abente, Mia K. Torchetti, Mary L. Killian, Amy L. Vincent

**Affiliations:** ^1^ Virus and Prion Research Unit Agricultural Research Service National Animal Disease Center U.S. Department of Agriculture Ames Iowa; ^2^ National Veterinary Services Laboratories, Veterinary Services U.S. Department of Agriculture Ames Iowa

**Keywords:** avian influenza, H7N9, influenza, LPAI, swine

## Abstract

In 2017, outbreaks of low and highly pathogenic avian H7N9 viruses were reported in four States in the United States. In total, over 270 000 birds died or were culled, causing significant economic loss. The potential for avian‐to‐swine transmission of the U.S. avian H7N9 was unknown. In an experimental challenge in swine using a representative low pathogenic H7N9 (A/chicken/Tennessee/17‐007431‐3/2017; LPAI TN/17) isolated from these events, no infectious virus in the upper and minimal virus in the lower respiratory tract was detected, nor was lung pathology or evidence of transmission in pigs observed, indicating that the virus cannot efficiently infect swine.

## 1 INTRODUCTION

In March 2017, highly pathogenic avian influenza (HPAI) and low pathogenic avian influenza (LPAI) A(H7N9) of North American lineage, distinct from the Asian lineage, were reported in poultry farms in Tennessee, USA.^1^ Subsequently, LPAI and HPAI H7N9 isolates were also detected in domestic poultry in three additional States: Georgia, Alabama, and Kentucky.^2^ The United States is the world's third largest producer and consumer of pork and pork products, and pig production systems are an important component of the agricultural economy of the United States.^3^ Geographic overlap between pig and poultry production systems and major migration flyways create a potential risk of an avian virus from wild birds spilling over into swine, as was recently reported with a wholly avian H4N6 virus isolated from a sick pig.^4^ Furthermore, avian‐origin genes have emerged and had sustained circulation among influenza A viruses (IAV) in swine.^5^, ^6^, ^7^ To assess the risk of this novel reassortant virus spilling over into the swine population, we experimentally challenged naïve and pre‐immune pigs with LPAI TN/17 to examine pathogenesis and transmission.

## 2 MATERIALS AND METHODS

### 2.1 Viruses and cell lines

The avian isolate (A/chicken/Tennessee/17‐007431‐3/2017; LPAI TN/17; GenBank Accession Numbers KY818816‐KY818823) was isolated from a broiler chicken breeder farm and confirmed by the USDA National Veterinary Service Laboratories as H7 LPAI. The LPAI TN/17 was passaged once in specific pathogen‐free embryonated chicken eggs inoculated by the allantoic sac route. The egg‐passaged stock was grown in Madin‐Darby canine kidney (MDCK) cells to prepare inoculum.

### 2.2 Animal study

Sixty‐five crossbred pigs were obtained from a healthy herd for the challenge study and split into naïve (Table 1) and pre‐immune groups (Table 2). For the naïve groups, two distinct age cohorts were used to evaluate whether age affected susceptibility to infection. On the day of challenge, fifteen pigs were 4 weeks old and twenty pigs were 14 weeks old. Pigs were confirmed to be seronegative to influenza A virus (IAV) antibodies against the nucleoprotein (NP) as measured by ELISA (Swine Influenza Virus Antibody Test, IDEXX, Westbrook, ME) prior to the study. Pigs from both age cohorts were divided into two groups: non‐challenged and challenged. Additionally, contact pigs were included in the 4‐week‐old cohort to examine transmission. For pre‐immune experiments, pigs were inoculated with 2 mL of 10^5^ TCID50/mL delivered intranasally at 4 weeks of age with either H1 (A/California/04/2009 H1N1 or A/swine/Minnesota/02011/2008 H1N2) or H3 (A/swine/Missouri/A01476459/2012 H3N2) viruses. Subsequent NP ELISAs confirmed that pigs challenged with these three viruses were seropositive prior to being challenged 33 days later with LPAI TN/17.

**Table 1:** Summary of virological and serological analysis of collected samples for naïve pigs

	Nasal Swabs^a^		Bronchoalveolar lavage fluid^b^		Serology	
4‐week‐old pigs	Virus isolation	Real‐time RT‐PCR	Virus isolation (TCID_50_/mL)	Real‐time RT‐PCR (C_T_ values)	NP ELISA 5 dpi/16 dpc	HI (≥ 10) 5 dpi/16 dpc
Challenged (10)	0/10	0/10	2/10	4/10 (24.6^c^, 25.9^c^, 32.1, 32.9)	0/10	0/10
Contact (5)	0/5	0/5	‐b	‐b	0/5	0/5
14‐week‐old pigs						
Challenged (10)	0/10	0/10	1/10	2/10 (29.0^c^, 30.7)	0/10	0/10

a Samples collected at 1‐5 dpi for primary challenged pigs and 1‐5, 7, and 9 dpc for contact pigs.

b BALF samples were not collected.

c Samples that were positive by virus isolation.

**Table 2:** Summary of virological and serological analysis of collected samples in pre‐immune pigs challenged with LPAI TN/17

	Nasal Swabs	Bronchoalveolar lavage fluid	Serology
14 weeks old at day of challenge with LPAI TN/17 (exposure virus)	Virus isolation^b^	Virus isolation (TCID_50_/mL)	HI positive (≥10)^c^ 5 dpi
Challenged (10) (A/California/04/2009 H1N1^a^)	0/10	0/10	0/10
Challenged (9) (A/swine/Minnesota/02011/2008 H1N2^a^)	0/9	0/9	0/9
Challenged (10) (A/swine/Missouri/A01476459/2012 H3N2^a^)	0/10	0/10	0/10

a Pigs exposed to 2 mL of 10^5^ TCID_50_/mL delivered intranasally at 4 weeks of age.

b Samples collected at 1‐5 dpi.

c HI assay performed using LPAI TN17 as the antigen.

Pigs challenged with LPAI TN/17 were inoculated simultaneously via intranasal (1 mL via slowly dripping 0.5 mL of virus into each nostril) and intratracheal (2 mL via laryngoscope and tracheal tube) routes with 10^5^ 50% tissue culture infective dose (TCID_50_) per mL of virus. Inoculation was performed while the pigs were under anesthesia by an intramuscular injection of a cocktail of ketamine (8 mg/kg of body weight; Zoetis Animal Health, Florham Park, NJ), xylazine (4 mg/kg), and tiletamine‐zolazepam (Telazol; 6 mg/kg) (Zoetis Animal Health, Florham Park, NJ). On 2 days post‐infection (dpi), five 4‐week‐old naïve contact pigs were placed in a separate raised deck in the same room as the ten 4‐week‐old pigs in the naive group that had been challenged, but with separate food and water and approximately 2 feet (0.6 m) away from the inoculated group to evaluate indirect contact transmission.

### 2.3 Sample collection and pathological examination of lungs

Nasal swab samples were collected daily 1‐5 dpi for challenged and negative control pigs, and 1‐5, 7, and 9 days post‐contact (dpc) for contact pigs. Negative control and challenged pigs were humanely euthanized at 5 dpi, when sera and bronchoalveolar lavage fluid (BALF) were collected. The lungs were evaluated, and the percentage of the surface of the entire lung affected by pneumonia was calculated on the basis of the weighted proportions of each lobe to the total lung volume. Five contact pigs were humanely euthanized at 16 dpc, and sera were collected to assess seroconversion.

### 2.4 Virus isolation and titers in nasal swab (NS) and BALF lung samples

Bronchoalveolar lavage fluid and NS samples (0.45 μm filtered) were plated onto confluent MDCK cells. 10‐fold serial diluted virus titrations were performed in triplicate on confluent MDCK cells in 96‐well plates starting with 10 μL of BALF fluid representing a 10^−2^ dilution. Virus isolation and viral titer assays were performed with 1 μg/mL tosylsulfonyl phenylalanyl chloromethyl ketone (TPCK) trypsin. After 48 hours, plates were fixed with 4% phosphate‐buffered formalin and stained using a mouse monoclonal antibody (H16‐L10‐4R5‐ clone HB65) to detect NP. While all serially diluted samples were initially deemed negative, RT‐PCR‐positive BALF samples (see below) were then subjected to virus isolation with 100 μL (10^−1^ dilution) placed on MDCK cells, resulting in propagation of three virus isolation‐positive samples (Table 1).

### 2.5 Nucleic acid extraction and real‐time RT‐PCR

RNA was extracted from NS and BALF samples with the MagMAX Viral RNA Isolation Kit (Thermo Fisher Scientific Inc, Waltham, MA). RT‐PCR was performed with the VetMAX‐Gold SIV Detection Kit following the manufacturer's instructions (Thermo Fisher Scientific Inc) with a Ct value of less than 35 deemed as definitively positive for influenza.

### 2.6 Serology

Sera were treated with receptor‐destroying enzyme (Denka Seiken, Japan), heat inactivated at 56°C for 30 minutes, and adsorbed with 50% turkey red blood cells (RBCs) to remove non‐specific hemagglutinin inhibitors and natural serum agglutinins. Hemagglutination inhibition assays were performed with TN/17 as the antigen and 0.5% turkey RBCs using standard techniques.

## 3 RESULTS

Pigs directly inoculated with LPAI H7N9 had no evidence of macroscopic lesions when compared to non‐infected control pigs (data not shown), and pre‐immune pigs were no different than naïve pigs in any parameter. Virological and serological results from collected samples are summarized in Table 1. In the challenged pigs, there was no evidence of infectious virus being shed through nasal secretions and only three animals had low levels of detectable virus in the lung. All of the sera samples were negative for HI activity and NP reactivity. Influenza RNA was detected by RT‐PCR in six of twenty inoculated pigs in the BALF samples from 5 dpi, but in none of the nasal swabs. Consistent with the absence of viral infection in challenged animals, contact pigs were also negative for virus and IAV‐specific antibodies. Samples from non‐challenged pigs (n* *=* *10 for each age group) were negative for virus isolation, and NP and HI titers.

## 4 DISCUSSION

Experimental challenge of pigs with a LPAI H7N9 isolate resulted in minimal replication in the lung with no evidence of transmission to contact pigs, suggesting a low risk to the swine population. Due to logistical challenges of studying the HPAI H7N9, we tested the LPAI H7N9 TN/17 isolate due to its genetic relatedness to the HPAI that was detected at the same time. The HA of the LPAI TN/17 isolate used in this study shares high homology with the HPAI H7N9 across the genome (see Figure S1, Table S1). H7N9 of other phylogenetic clades have caused five epidemic waves in humans in China since 2013 and remain a public health concern.^8^ Notably, Asian LPAI H7N9 has caused the majority of human infections with most human isolates having acquired G186V and Q226L/I substitutions in the HA and E627K substitution in the PB2,^8^, ^9^ although HPAI H7N9 remains a risk to the human population.^10^ The LPAI TN/17 tested in this study is of North American wild bird lineage and does not possess these previously identified adaptive substitutions.

Three pig challenge studies found that early human isolates from the first wave of the H7N9 epidemic in China, A/Shanghai/02/2013 and A/Anhui/1/2013, caused mild disease although infectious virus was isolated from nasal swab samples.^11^, ^12^, ^13^ Both of these human isolates encode mammalian adaptive substitutions in the HA (Figure S1). In one study, a control avian LPAI A/chicken/Zhejiang/DTID‐ZU01/2013 H7N9 was used that has the G186V substitution although it encodes 226Q in the HA and virus shedding was observed. Furthermore, serially passaged A/Anhui/1/2013 acquired a L226Q change in pigs, suggesting that G186V may be more important for infection in pigs.^12^

Belser et al^2^ found that ferret infections with both LPAI and HPAI were restricted to the upper respiratory tract, but nasal titers were positive in only one of three contact ferrets for LPAI TN/17 while there was no contact transmission with the HPAI. In BALB/c mice, both LPAI and HPAI infections resulted in mild illness and no deaths. In contrast to these animal studies, we found that the LPAI TN/17 was restricted from efficiently replicating in pigs, even in the upper respiratory tract where ferrets were susceptible. We also examined swine susceptibility to LPAI TN/17 in pre‐immune pigs exposed to H3 and H1 viruses and did not detect any differences in protection or susceptibility similar to the naïve pigs.

Our results, in combination with the published ferret and mouse studies,^2^ indicate that the 2017 H7N9 poultry virus reported in the United States would likely require further adaptation to infect and be sustained in a mammalian host. Nonetheless, there is potential for mutation or reassortment to acquire swine‐origin genes for adaptation. Introduction of a novel HA into the swine population would have a profound effect on the antigenic landscape of circulating strains in pigs and potentially pose a risk of spilling over into the human population. Continuous swine surveillance is paramount for early detection of spillover events, and controlled challenge experiments remain an important risk assessment tool to help identify potential threats to animal and public health.

## CONFLICT OF INTEREST

The authors declare no conflict of interest.
