# Supplementary figures and images for: An avian influenza virus A(H7N9) reassortant that recently emerged in the United States with low pathogenic phenotype does not efficiently infect swine
Source: Influenza Other Respir Viruses. 2019 Feb 13;13(3):288–91. doi: 10.1111/irv.12631 (PMC6468088; doi:10.1111/irv.12631)

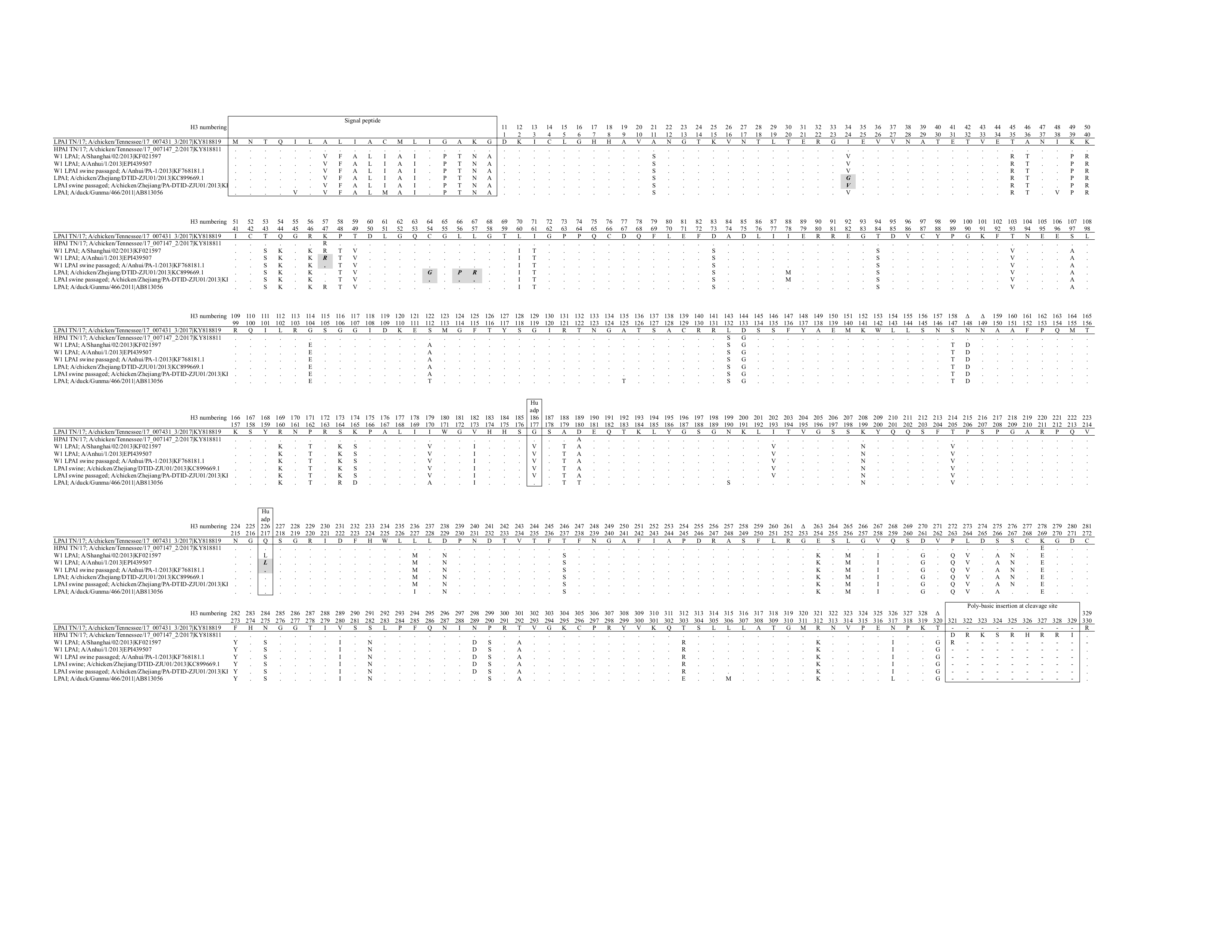

Supplement: Supplementary file 1 [file IRV-13-288-s001.jpg]
