# Supplementary material for: An avian influenza virus A(H7N9) reassortant that recently emerged in the United States with low pathogenic phenotype does not efficiently infect swine
Source: Influenza Other Respir Viruses. 2019 Feb 13;13(3):288–91. doi: 10.1111/irv.12631 (PMC6468088; doi:10.1111/irv.12631)
Supplement: Supplementary file 2 [file IRV-13-288-s002.docx]

| **Table S1.** Amino acid comparison of H7N9 LPAI TN/17 A/chicken/Tennessee/17-007431-3/2017 and HPAI TN/17 A/chicken/Tennessee/17-007147-2/2017. | | | |
| --- | --- | --- | --- |
| Protein | % identity of  LPAI with HPAI | Number of amino acid differences  LPAI vs. HPAI | Amino acid differences  LPAI vs. HPAI |
| HA | 97.5 | 14^a^ | ^b^ K57R, L143S, D144G, D189A, K278E, poly-basic insertion at cleavage site: DRKSRHRRI |
| NA | 99.8 | 1 | D357N |
| PB1 | 99.7 | 2 | K13P, H394P |
| PB2 | 99.6 | 3 | K8R, H392Q, T637I |
| NP | 99.8 | 2 | M105I, F478S |
| NS1  NS2  M1  M2 | 98.7  99.2  100  100 | 3  1 | W124M, I155V, Q193R  K36E |

^a^ Includes 9 amino acid insertion at cleavage site not found in LPAI

^b^ H3 numbering
